# Carbonyls Mediated Dual‐Function Enables High‐Performance Carbon Anodes in Ester‐based Electrolyte

**DOI:** 10.1002/advs.202503954

**Published:** 2025-06-20

**Authors:** Ziyu Wu, Fei Yuan, Siyu Wu, Di Zhang, Qiujun Wang, Qujiang Sun, Zhaojin Li, Wei Wang, Bo Wang

**Affiliations:** ^1^ Hebei Key Laboratory of Flexible Functional Materials School of Materials Science and Engineering Hebei University of Science and Technology Shijiazhuang 050000 China; ^2^ School of Metallurgical and Ecological Engineering University of Science and Technology Beijing Beijing 100083 China

**Keywords:** hard carbon, interfacial stability, potassium ion battery, surface reconstruction

## Abstract

Constructing an “ether solid electrolyte interphase (SEI)”‐like layer in ester electrolyte is highly attractive to realize excellent capacity and cycling stability for hard carbon anode, but has barely been mentioned yet. Herein, the hard carbon grafted by caffeic acid is developed via a surface reconstruction strategy, in which rich C═O moieties are introduced. Varied characterizations reveal that the existing C═O exhibits stronger adsorption energy on salt (PF_6_^−^) than solvent (e.g., EC and DEC), thus accelerating salt decomposition to produce a stable inorganic SEI layer. Besides, C═O moieties can also adsorb K‐ions reversibly, accounting for a high capacitive contribution. Benefiting from the double merits of C═O moieties, the interfacial stability and surface properties of the optimized sample are greatly improved, and as a result, the reversible capacity can reach 462.7 mAh g^−1^ (0.1 A g^−1^ over 50 cycles) and rate performance is quite superior as well (321.8 mAh g^−1^ at 2 A g^−1^). Besides, a prolonged cycle life of over 2000 cycles is smoothly realized at 2 A g^−1^ in ester‐based electrolytes. This work provides insight into the electrochemical performance improvement of ester‐based electrolytes via structure design.

## Graphical abstract

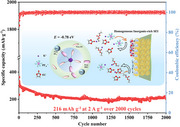

## 1 Introduction

Potassium ion batteries (PIBs) are considered as a sustainable technology for large‐scale energy storage and smart grids due to their low cost and excellent ion migration kinetics in electrolytes.^[^ ^1^, ^2^ ^]^ Not only that, the redox potential of K/K^+^ (−2.93 V vs standard hydrogen electrode) is close to that of Li/Li^+^ (−3.04 V), enabling a high energy density and working potential in the full‐cell.^[^ ^3^, ^4^ ^]^ However, given a larger K^+^ size (0.138 nm), the most commonly used anode materials usually exhibit slow bulk‐phase diffusion kinetics and serious structural degradation upon repeated dis/charging process,^[^ ^5^, ^6^ ^]^ thus leading to poor rate performance and cycling stability. This conclusion is well supported by a fact, namely the volume expansion of graphite anode can reach 60% when full potassiation, and its narrow interlayer spacing (0.335 nm) is unfavorable for ion diffusion and buffer structure stress, so the rate capability hardly reaches 175 mAh g^−1^ even at a low current density, accompanied by cycling stability below 300 cycles.^[^ ^7^ ^]^ As a result, designing carbon anode with a better rate and high cycling stability is highly desirable for PIBs.^[^ ^8^, ^9^ ^]^

Unlike graphite, hard carbon consisting of more disordered structures can provide rich defect sites to store K^+^ via surface adsorption, which greatly reduces the structure deformation caused by carbon layer intercalation, enabling improved rate and cycling performance.^[^ ^10^, ^11^ ^]^ However, the existing defect sites easily cause the excessive depletion of ester‐based electrolyte solvent such as ethylene carbonate (EC) and diethyl carbonate (DEC), via some side reactions, leading to thick, uneven, and organic‐species enriched solid electrolyte interphase (SEI) layer.^[^ ^12^, ^13^ ^]^ As a result, the capacity of hard carbon in ester‐based electrolytes is poor, and a thick SEI inhibits the fast migration of charge, damaging rate capability at a high current density. Currently, the use of ether‐based electrolytes can help to form a SEI layer dominated by inorganic species (e.g., KF) on the basis of K‐salt preferential decomposition,^[^ ^14^, ^15^ ^]^ which not only accelerates ion diffusion through SEI but improves interfacial stability, favoring a high capacity. While effective in forming inorganic‐rich SEI layers, their high production costs and incompatibility with high‐voltage cathodes limit practical applications.^[^ ^16^, ^17^, ^18^ ^]^ The formation of inorganic‐rich SEI membranes has been achieved by electrolyte engineering, where high or locally high concentrations of electrolyte enable anion‐enhanced solvation to form inorganic‐rich SEIs.^[^ ^19^, ^20^ ^]^ In general, inorganic‐rich interfaces have stronger mechanical strength than organic‐rich interfaces, contributing to superior cycling stability performance. The introduction of 1,2‐dibutoxyethane (DBE) into the electrolyte can significantly change the interfacial dynamics and the embedding behavior of potassium ions, thus enhancing the cycling performance of potassium ions.^[^ ^21^ ^]^ Although 4‐Fluoro‐1,3‐dioxolan‐2‐one (FEC) additives enhance SEI stability, they induce severe anodic polarization, compromising interfacial structural stability at low potentials.^[^ ^22^ ^]^ Surface modification of hard carbon anode materials can also enhance the interfacial stability of SEIs. The B─OH group in polyether block polyamide (DEBA) reacts with ─COOH and ─OH on hard carbon to form a stable chemical bond by dehydration, and DEBA can be tightly connected with the hard carbon surface, and modify the hard carbon anode materials. DEBA relies on electrostatic interactions for K⁺ adsorption, offering limited capacity enhancement, while its long‐chain structure hinders ion diffusion.^[^ ^23^, ^24^ ^]^ In contrast, Caffeic acid (CA) grafting addresses these limitations synergistically, the ‐COOH groups in CA covalently bind to hard carbon via dehydration condensation, ensuring stable surface modification. Moreover, the C═O functional group has a dual function, first, stronger adsorption of K salts (relative to the solvent) to catalyze the formation of inorganic SEIs rich in KF, and second, reversible K⁺ adsorption to enhance the capacitance contribution. Based on this, HCS was modified by a surface reconstruction strategy to introduce C═O functional groups. Constructing a “ether‐SEI”‐like layer in ester electrolyte to realize high capacity is of great significance, but has barely been mentioned up to now.

Herein, based on a surface re‐construction strategy, hollow mesoporous carbon spheres with numerous C═O moieties are developed. Systematic characterizations demonstrate that the presence of C═O presents a double‐functional behavior. On the one hand, C═O has a stronger affinity to K‐salt than solvent molecules, thus catalyzing the preferential decomposition of K‐salt. As such, the SEI featured with rich KF species is formed on the surface of carbon, which improves interfacial K^+^ transfer and structural stability. On the other hand, C═O species can function as active central to reversibly adsorb K^+^, enabling an excellent capacitive contribution. All these together endow the optimized electrode with an ultra‐high capacity of 462.7 mAh g^−1^ and a quite superior rate performance (321.8 mAh g^−1^ at 2 A g^−1^). Also, a long cycle lifespan is delivered at 2 A g^−1^ over 2000 cycles with a capacity of 216 mAh g^−1^. The design route in this work opens a new avenue to realize high‐capacity carbon anodes in ester‐based electrolytes based on electrode structure tailoring.

## 2 Results and Discussion

**Figure 1:**
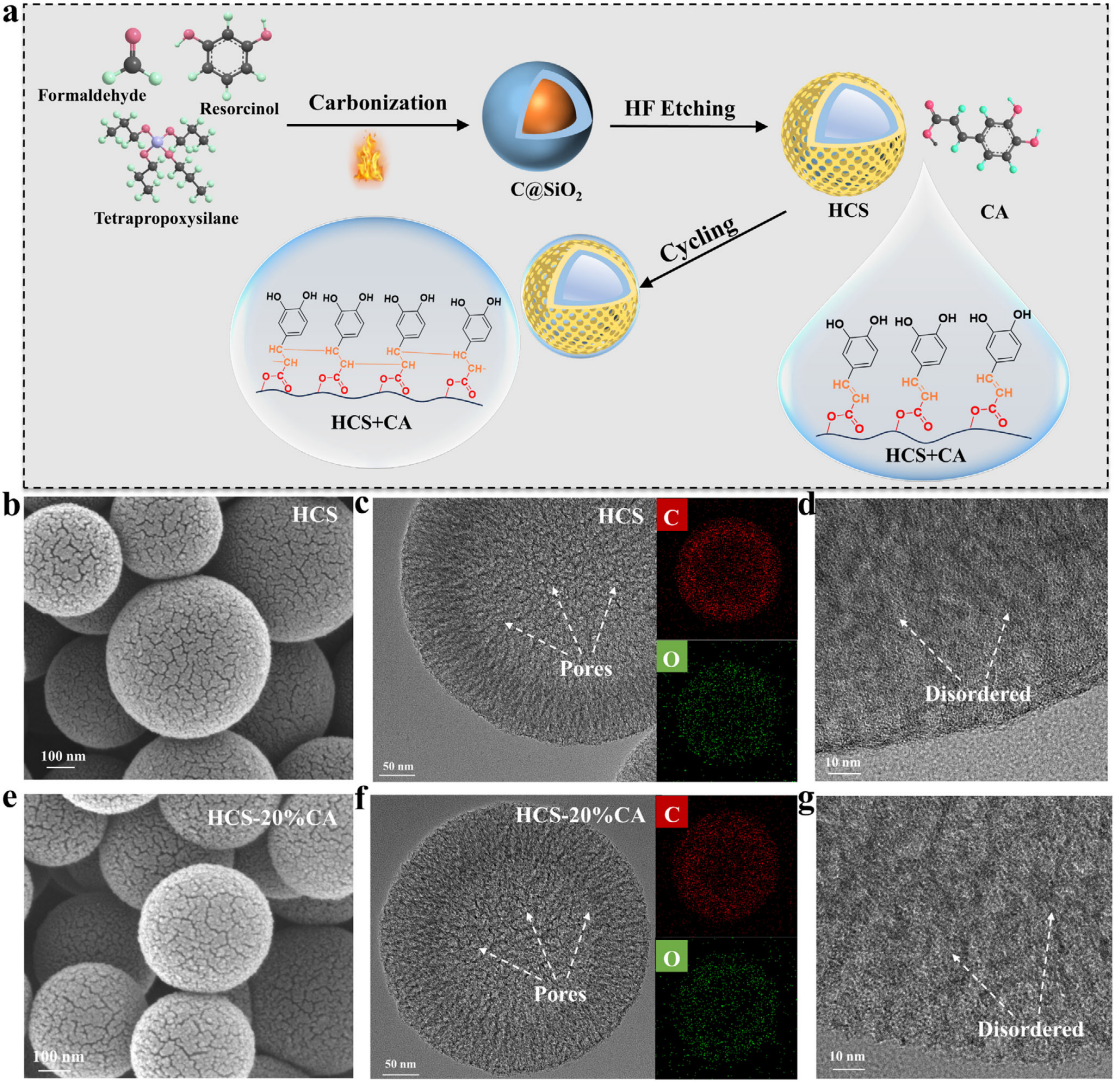
a) Schematic diagram of preparation of by grafting poly‐CA to regulate the interface chemistry of HCS anodes. b) SEM, c) TEM and corresponding element mappings of, and d) HRTEM images of HCS. e) SEM, f) TEM, and corresponding element mappings of and (g) HRTEM images of HCS‐20%CA, respectively.

The preparation process of C═O species grafted hollow mesopores hard sphere is schematically illustrated in **Figure** **1**a. Briefly, hollow mesoporous carbon spheres (HCS) were first prepared using SiO_2_ as a hard template, and then grafted with caffeic acid (CA) on their surface. Due to the dehydration condensation between ─COOH and ─OH, CA could be stably bonded to the surface of HCS (HCS‐20%CA), thus abundant C═O functional groups were introduced. As comparison, HCSs with different CA content were also constructed (more details are provided in Supporting Information), corresponding to HCS‐10%CA and HCS‐30%CA.

The morphological and structural properties of the materials were analyzed using SEM. As observed in Figure 1b,e, it is found that both HCS and HCS‐20%CA have a spherical morphology. Moreover, there are obvious pores in HCS and HCS‐20%CA (Figure 1c,f,), and they are mainly composed of C and O elements, while HCS‐20%CA has a higher O content (Figure S1, Supporting Information). As shown in Figure 1d,g, based on high‐resolution transmission electron microscopy (HRTEM) images of HCS and HCS‐20%CA, it is evident that they are composed of disordered structures. These observations demonstrate that the introduced CA has little effect on morphology and microstructure, and only causes the increase in O content. According to the SAED test (Figure S2, Supporting Information), it can be seen that the diffraction ring of (101) of the HCS sample is more obvious than that of the sample (101) of the HCS‐20%CA, indicating that the HCS sample has a higher degree of graphitization, while the HCS‐20%CA has more amorphous structures, and that the spacing of the carbon layers becomes larger after the introduction of the caffeic acid, which is more favorable for the transport of K^+^.

**Figure 2:**
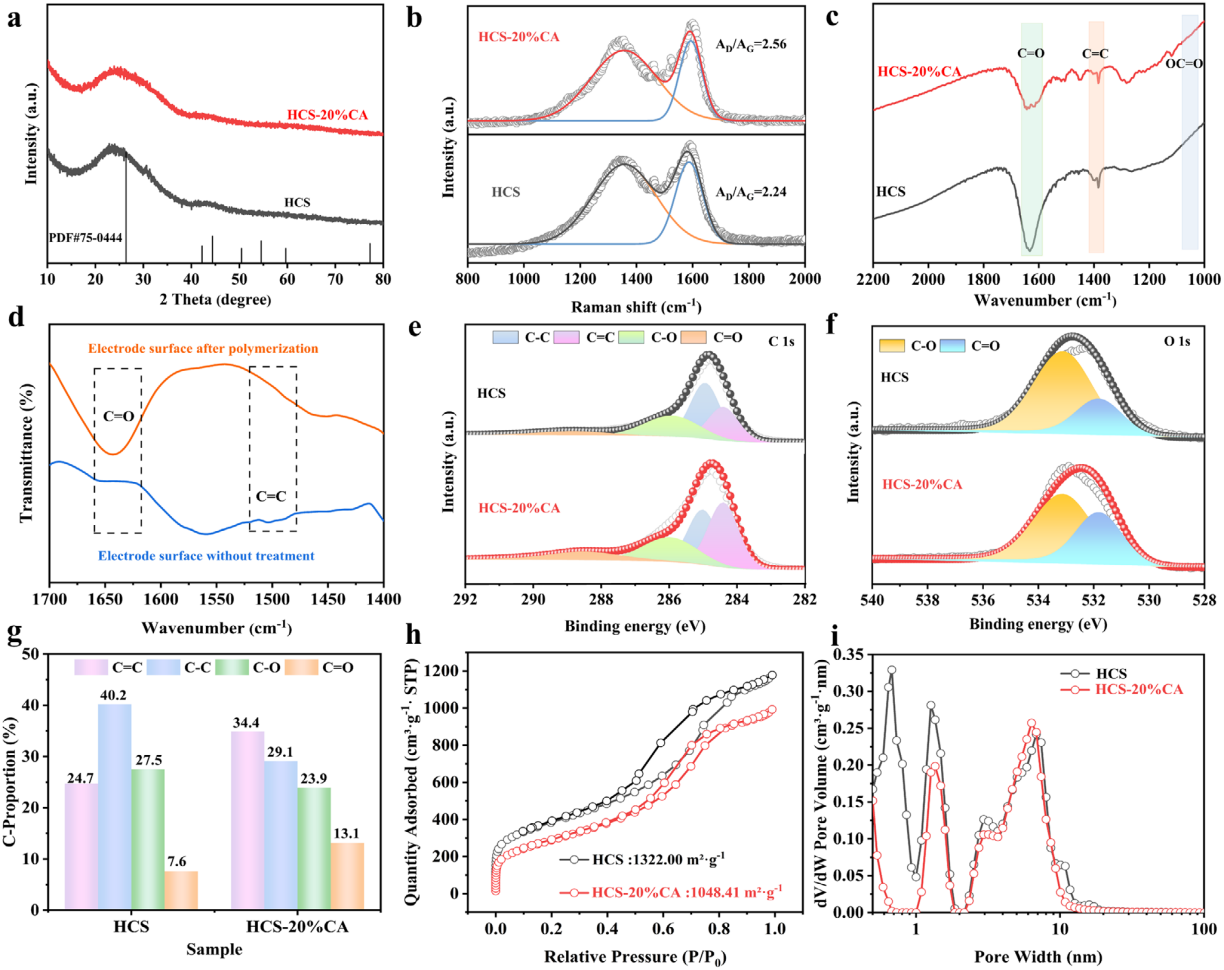
a) XRD patterns b) Raman spectra and c) FTIR spectra of HCS and HCS‐20%CA. d) FTIR spectra of the HCS‐20%CA anodes before and after discharge at a constant potential and e) high resolution C 1s f) O 1s spectra of HCS and HCS‐20%CA. g) C═C, C─C, C─O, and C═O proportion of HCS and HCS‐20%CA. h) N_2_ adsorption–desorption isotherms based on BJH modeling and i) pore size distribution of HCS and HCS‐20%CA based on DFT modeling.

The XRD patterns in patterns in **Figure** **2**a and Figure S3 (Supporting Information) illustrate that the presence of CA can slightly increase the disordered degree of carbon matrix, as evidenced by a lower (002) peak intensity of HCS‐20%CA than HCS. From Figure 2b and Figure S4 (Supporting Information), it is easy to see that the use of CA can increase defects, as evidenced by an increased A_D_/A_G_ value (2.56) in HCS‐20%CA, and the higher CA content, the more defects, which is consistent with the XRD results. The increased defects may be a result of the increased O‐content caused by more CA amount.^[^ ^25^, ^26^ ^]^ As shown in Figure S5 (Supporting Information), the Fourier transform infrared (FTIR) spectra of CA‐grafted HCS show several peaks that represent the CA feature, indicating the successful interconnection between HCS and CA.^[^ ^27^, ^28^ ^]^ Besides, the HCS has more C═O moieties after CA grafting, especially HCS‐20%CA displays an obvious C═O (1645 cm^−1^) peak, as depicted in Figure 2c. To demonstrate the successful grafting of CA onto HCS, HCS‐20%CA powder was processed into electrodes to assemble a PIBs half‐cell. During discharge, electrons transfer from HC to the unsaturated C═C (1440 cm^−1^) bond of CA. The process subsequently initiates chain growth and promotes the anionic polymerization of CA molecules, resulting in the formation of polymeric films. As evidenced by the FTIR spectra of the electrodes prior to discharge (Figure 2d), distinct C═C bonds are observed, which significantly weaken after discharging. This confirms the cleavage of C═C bonds and their subsequent participation in polymerization reactions. The surface chemical and element composition were further investigated by XPS measurements (Figure S6, Supporting Information). Evidently, all the samples are composed of C and O elements, and the introduced CA induces the increase in O‐content. Through Figure 2e and Figure S7a,b (Supporting Information), it is found that the C 1s of all the samples can be divided into four peaks at 284.3, 284.8, 285.8, and 288.5 eV, corresponding to C═C, C─C, C─O, and C═O, respectively. The high‐resolution O 1 s spectra (Figure 2f and Figure S7c,d, Supporting Information) is deconvoluted into C─O and C═O peaks at 533.1 and 531.8 eV, respectively. Combined with Figure 2g, Figures S8 and S9 (Supporting Information), one can see that C═O species content first increases and then decreases with increasing CA addition amount, so HCS‐20%CA realizes the highest C═O content, namely 38.4%. The pore structure was studied by N_2_ de/adsorption measurements, and the associated findings are illustrated in Figure 2h. The pore structure was studied by N_2_ de/adsorption measurements, and the specific surface area was quantified by Barret–Joyner–Halenda (BJH) modeling, and the specific surface area (SSA) of HCS decreased from 1322.00 to 1048.41 m^2^ g^−1^ after grafting CA, which could be attributed to the covering effect of CA on micropores. The pore structure was quantified by density functional theory (DFT) modeling (Figure 2i), and the pore size distribution confirmed this result, with a slight decrease in the microporous volume of HCS‐20% CA, while maintaining the abundant mesopores.

**Figure 3:**
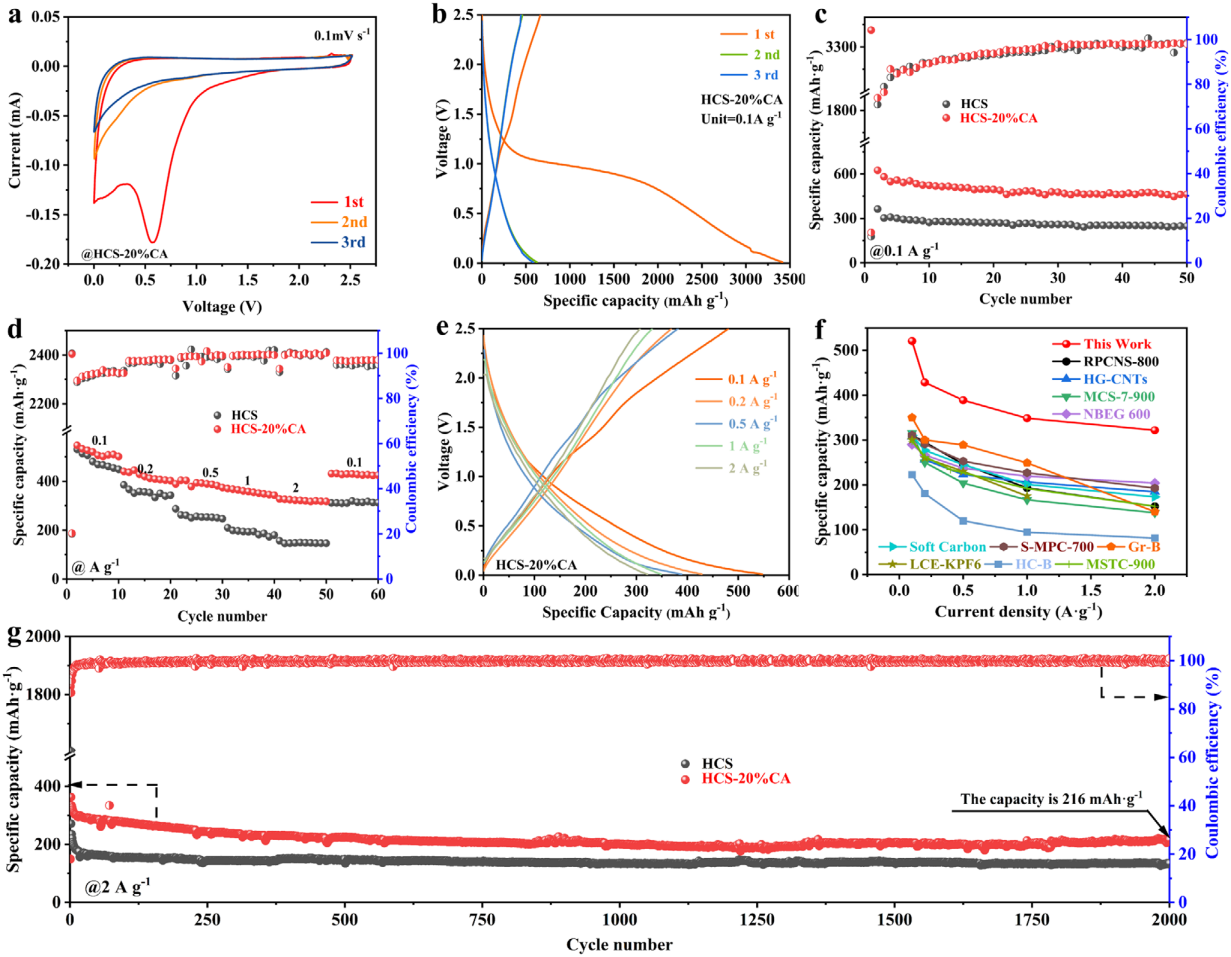
Electrochemical performance characterization of resulting products. a) CV curves at 0.1 mV s^−1^ and b) the initial three dis/charging curves at 0.1 A g^−1^ for HCS‐20%CA. c) Cycling performance and stability of HCS‐20%CA at 0.1 A g^−1^. d) Comparison of cycling performance at 0.5 A g^−1^ and e) dis/charging curves of HCS‐20%CA at varied current density. f) Comparison of rate performance of HCS‐20%CA and representative carbon materials. g) Long‐cycling stability of HCS‐20%CA at 2 A g^−1^.

The potassium storage behavior of HCS‐20%CA was first investigated using CV measurements. **Figure** **3**a displays the first three CV curves recorded at 0.1 mV s^−1^. A distinct reduction peak observed near 0.6 V during the first cycle is attributed to electrolyte decomposition and the formation of the solid electrolyte interphase (SEI) layer. This peak is absent in the subsequent cycles, indicating the formation of a stable and uniform SEI layer.^[^ ^29^, ^30^ ^]^ Through Figure 3b and Figure S10 (Supporting Information), the initial Coulombic efficiency (ICE) of HCS‐20%CA is 19.5%, which is higher than that of HCS (12.8%), HCS‐10%CA (13.5%), and HCS‐30%CA (14.1%). It is worth noting that HCS‐20%CA has a similar SSA to HCS but more defects, so its high ICE likely results from more C═O moieties that optimize the formation of SEI (discussed later). However, the ICE of HCS‐20%CA is too low to achieve the practical application in terms of energy density and capacity, so that pre‐potassiation or electrolyte design needs to be used before assembling a full cell. When cycled at 0.1 A g^−1^ (Figure 3c), HCS‐20%CA exhibits an ultra‐high capacity of 462.7 mAh g^−1^, which is superior to its counterparts. Even when the current density is raised to 0.5 A g^−1^ (Figure S11, Supporting Information), HCS‐20%CA maintains a capacity of 325.6 mAh g^−1^ after 50 cycles. The rate capability of HCS‐20%CA is further illustrated in Figure 3d, with average capacities of 501.4, 406.9, 393.7, 351.5, and 321.8 mAh g^−^¹ achieved at current densities of 0.1, 0.2, 0.5, 1, and 2 A g^−1^, respectively. In comparison, HCS demonstrates a reduced capacity, especially under high current conditions of 2 A g^−^¹, its capacity is only 149.5 mAh g^−1^. Compared to HCS (Figure S12, Supporting Information), the dis/charging curves shape of HCS‐20%CA (Figure 3e) at different current densities has almost no changes, highlighting an excellent rate performance. Comparatively, HCS‐20%CA has more defects and C═O species compared to HCS, so its excellent rate can be ascribed to increased ion diffusion ability based on adsorption behavior. However, the rate capability is also determined by interfacial kinetics related to the SEI layer, and combined with ICE analysis, it can be learned that HCS‐20%CA has a more ideal interfacial. Benefiting from the double advantages of C═O functional groups, the rate performance of HCS‐20%CA also markedly outperforms some previous studies,^[^ ^13^, ^31^, ^32^, ^33^, ^34^, ^35^, ^36^, ^37^, ^38^, ^39^ ^]^ as shown in Figure 3f. The cycling stability of HCS‐20%CA was demonstrated in Figure 3g, in which HCS‐20%CA can experience 2000 cycles at 2 A g^−1^ via keeping a high capacity of 216 mAh g^−1^. Although HCS also undergoes 2000 cycles, its reversible capacity is much lower than 200 mAh g^−1^. It should be pointed out that the cycling stability of carbon anode in ester‐based electrolytes is quite poor, resulting from the formation of organic‐rich SEI caused by the excessive decomposition of solvent. Therefore, the observed excellent cycling stability of HCS‐20%CA can be ascribed to the change of SEI formation, once again emphasizing the great effect of C═O on SEI. In this context, the cycle performance of HCS‐20%CA is superior to most previously reported work, as evidenced by Table S1 (Supporting Information).

**Figure 4:**
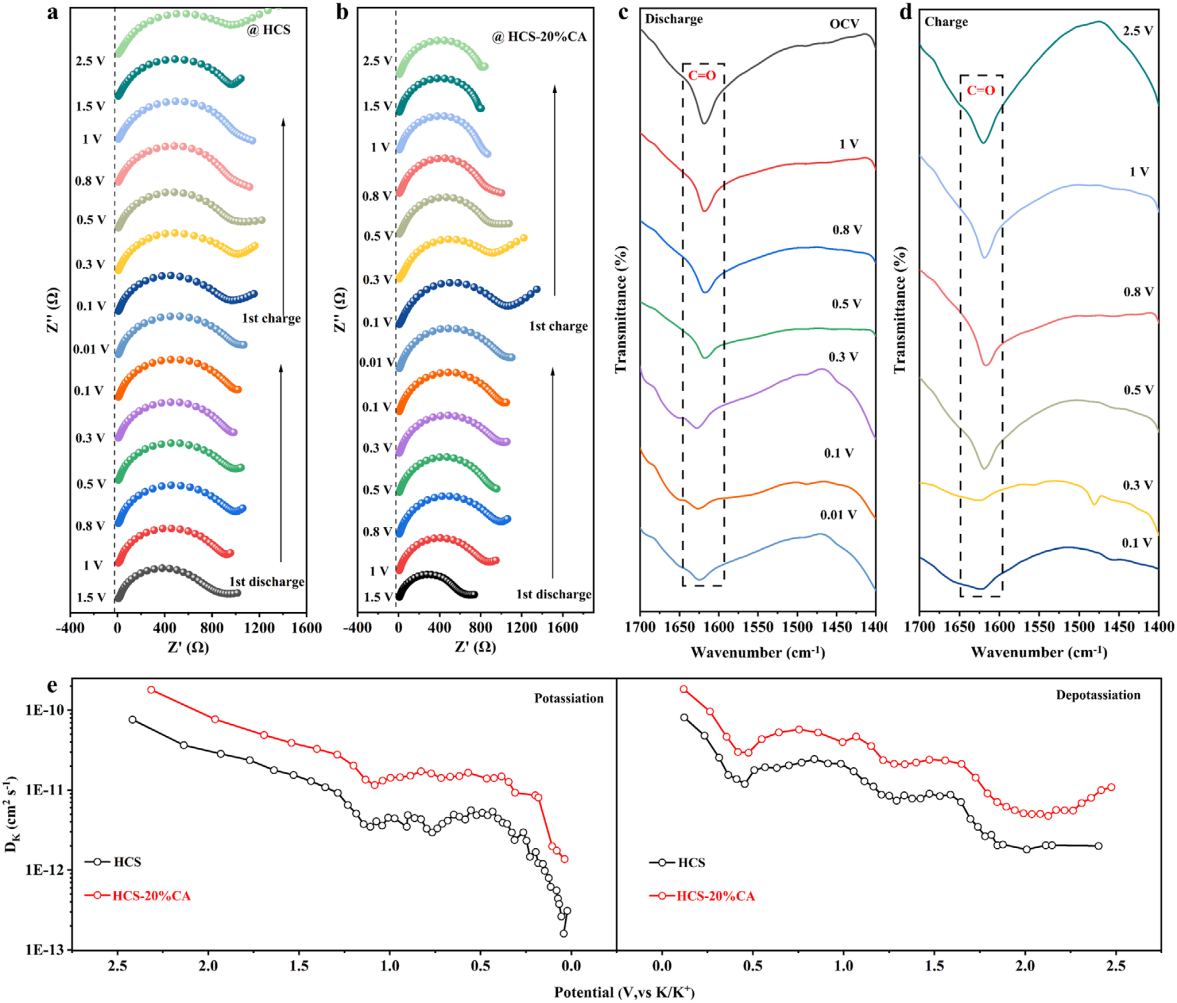
Nyquist plots of a) HCS and b)HCS‐20%CA at different potentials during dis/charging processes. c,d) FT‐IR spectra of HCS‐20%CA at different potentials during galvanostatic charge/discharge processes. e) D_k_ values of HCS and HCS‐20%CA.

As mentioned above, HCS‐20%CA featuring rich C═O functional groups exhibits better capacity, rate, and cycling stability than HCS. Therefore, the charge transfer kinetics of HCS‐20%CA and HCS electrodes were investigated via in situ electrochemical impedance spectroscopy (EIS). Based on the selected potential (Figure S13a,b, Supporting Information), the tested results are provided in **Figure** **4**a,b. Note that the semicircle in the high/mid‐frequency region represents the charge transfer resistance (Rct), which indirectly reflects the resistance of the SEI membrane in literature.^[^ ^40^ ^]^ On the contrary, the slope of the diagonal line in the low‐frequency range represents the Warburg impedance of ion diffusion.^[^ ^19^ ^]^ Nyquist plots were fitted using an equivalent circuit model to analyze the change in resistance (Figure S14, Supporting Information). Compared to HCS, in the initial discharge process of HCS‐20%CA, namely, from the open‐circuit voltage (OCV) to 0.8 V, the Rct gradually increases with the gradual embedding of K^+^ into the electrode, indicating the formation of SEI film. As the discharge process proceeds (0.8–0.5 V), the Rct of HCS‐20%CA decreases, unveiling the formed SEI is more conductive. Besides, the Rct in the voltage range of 0.8–0.01 V is almost constant, indicating that the formed SEI membrane is stable and dense, and hence inhibits the continuous decomposition of electrolyte. All of these intuitively demonstrate that rich C═O moieties positively accelerate the formation of ideal SEI, leading to a faster ion diffusion ability. Not only that, the effect of C═O on K‐ion storage was also evaluated based on ex situ FTIR. As shown in Figure 4c,d, the intensity of C═O peak obviously decreases when the potential is above 0.5 V, while that slightly decreases in the range of 0.5–0.01 V, meaning that C═O moieties mainly adsorb K‐ion in the high potential region. This adsorption is highly reversible, due to the recovered C═O (1620 cm^−1^) peak after full charging to 2.5 V. Combining with EIS and FTIR, it is illustrated that C═O has a double functional behavior, namely optimizes SEI formation and adsorbs K‐ions, thus leading to improved ion migration kinetics. In order to evaluate the effect of K^+^ adsorption on hard carbon materials after grafting CA, the binding energy of K^+^ on the HCS surface was evaluated by density functional theory(DFT) simulation, as shown in Figure S15 (Supporting Information), the adsorption energy (∆E)of the defect‐rich graphene material was −2.14 eV, whereas ∆E of the HCS‐20%CA was −2.74 eV after the introduction of the C═O functional groups through the grafting of CA. This enhancement directly demonstrates that the C═O moieties, when integrated into a defect‐rich carbon framework, exhibit stronger interactions with K⁺ ions compared to unmodified systems.

To investigate the potassium storage kinetics, the CV measurements at multiple scan rates and GITT were employed. The CV test results (Figure S16a,b, Supporting Information) showed that the value of the slope b could be fitted according to the equation

(1) lgi=lga+blgv

The values of b were 0.84 and 0.88 for HCS and HCS‐20% CA, respectively (Figure S16c, Supporting Information), suggesting that the potassium storage mechanism is contributing to both capacitive and diffusive behaviors. As can be seen from Figure S16d (Supporting Information), the capacitance contribution of HCS‐20% CA is significantly higher than that of HCS, which suggests that the C═O group can reversibly adsorb K ions, resulting in a higher capacitance contribution. In this study, the diffusion capacity of 0.5 A g^−1^ was calculated by determining the diffusion contribution of CA grafted samples with different concentrations at 0.1 mV s^−1^ (Figure S17a, Supporting Information) and correlating it with the content of C═O functional group (Figure S17b, Supporting Information). The results showed that the adsorption capacity increased with the increase of the content of C═O functional group, which proved that the content of C═O functional group contributed to the adsorption capacity. This opinion is further supported by the GITT test result. According to Figures S18 and S19 (Supporting Information), the calculated K^+^ diffusion coefficients (D_k_) values are presented in Figure 4e, it is evident that HCS‐20% CA always has a higher D_k_ than HCS during the discharge/charge process.^[^ ^41^, ^42^ ^]^

**Figure 5:**
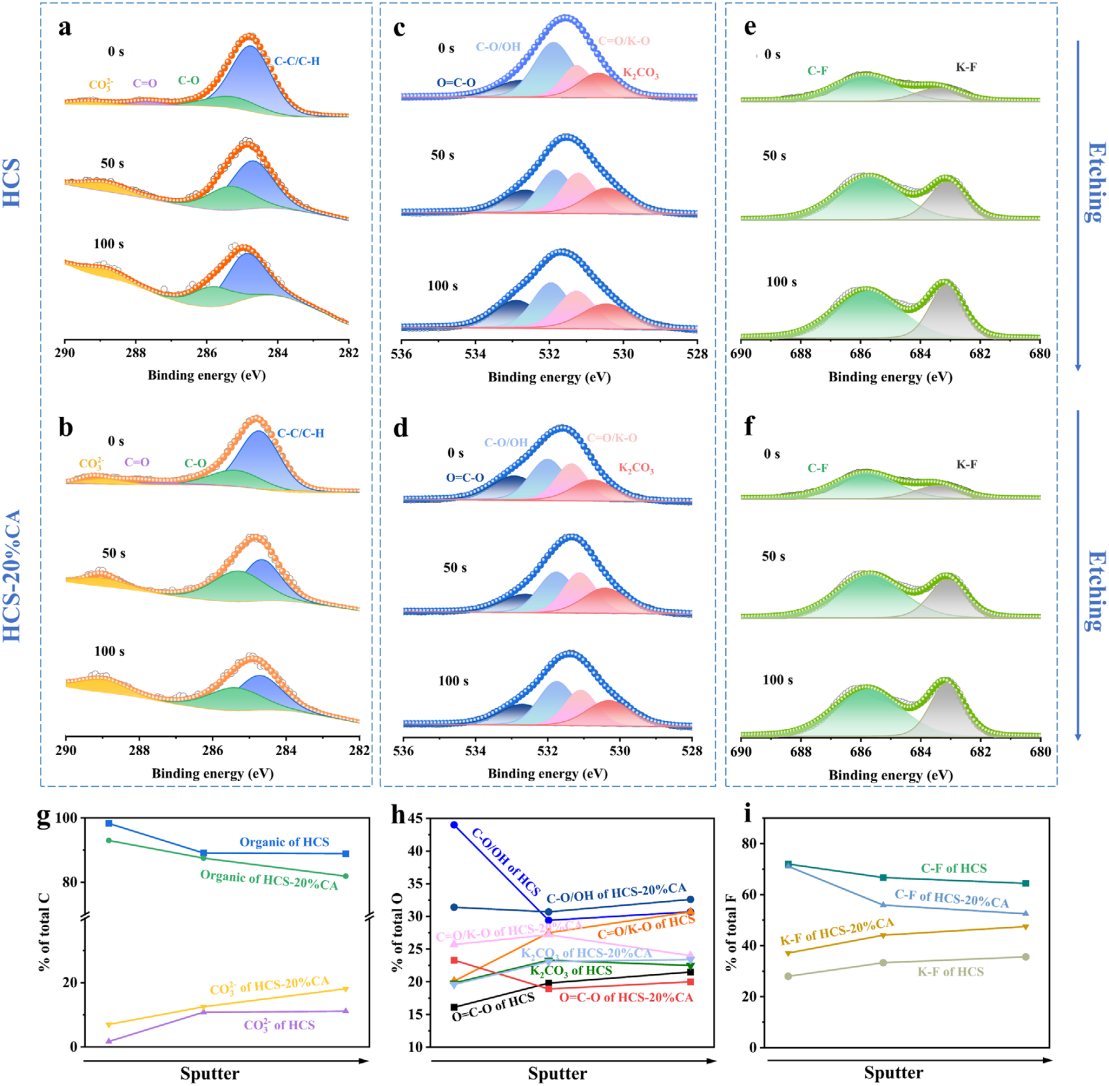
Etching XPS spectra of a,b) C 1s, c,d) O 1s and e,f) F 1s of SEI on the HCS and HCS‐20%CA. The proportion of SEI components is calculated from the g) C 1s, h) O 1s, and i) F 1s spectra.

To further validate the SEI evolution mechanism, we conducted atomic force microscopy (AFM) on cycled electrodes to characterize the surface morphology and roughness of the SEI layer^[^ ^43^ ^]^. The AFM results (Figure S20, Supporting Information) reveal that the HCS‐20%CA electrode exhibits a significantly smoother surface (Ra = 0.114 µm) compared to the pristine HCS electrode (Ra = 0.225 µm). The 3D topographic images further demonstrate that the SEI on HCS‐20%CA displays a homogeneous and compact structure, whereas the SEI on HCS shows irregular block‐like features with pronounced roughness. To investigate the structural and compositional characteristics of the SEI layer influenced by C═O functional groups, the cycled electrode was analyzed using XPS at varying etching durations. Although its accuracy is not enough compared to some in situ characterizations. These observations align with the XPS depth‐profiling results (**Figure** **5**), confirming that the C═O‐mediated SEI on HCS‐20%CA achieves superior uniformity and a balanced organic/inorganic composition. Through precise analysis of XPS spectral peaks and their corresponding binding energies, it was determined that the SEI components are essentially similar, (Tables S2 and S3, Supporting Information) prior to sputtering (Figure 5a–f), and several peaks observed in C 1s (C─C/C─H, C─O, and C═O/COO─) and O 1s (─C─O, C─O/O─H, and C═O) can be ascribed to the organic matter ROCO_2_K/(CH_2_OCO_2_K)_2_, which originates from the decomposition of the solvent. In addition, CO_3_^2−^ in C 1s, K─O in O 1s, and K─F in F 1s can be attributed to K_2_CO_3_, K_2_O, and KF inorganic components, respectively,^[^ ^15^ ^]^ where K_2_CO_3_/K_2_O is generated through the reaction of K^+^ with decomposed solvent by‐products, and K─F originates from the decomposition of KPF_6_ salts.^[^ ^12^ ^]^ The presence of a C─F bond, identified in the F 1s spectrum of HCS‐20%CA, indicates the incorporation of fluorinated organic compounds within the SEI layer.^[^ ^28^, ^44^ ^]^ These compounds play a crucial role in suppressing the dissolution of SEI components and enhancing electrochemical stability. As sputtering time increases, the SEI of HCS exhibits a greater content of organic constituents compared to HCS‐20%CA (Figure 5g,h), suggesting a higher degree of solvent decomposition on the HCS electrode. In contrast, HCS‐20%CA demonstrates a significantly higher proportion of inorganic components, particularly K─F compounds, as illustrated in Figure 5i. As summarized in Table S4 (Supporting Information), the HCS‐20%CA anode retained significantly higher C═O concentrations (25.7% post‐cycling) compared to the HCS anode (20.1% post‐cycling). This minimal attenuation of C═O content in HCS‐20%CA unequivocally demonstrates the robust retention of CA‐derived moieties during prolonged electrochemical cycling, further confirming the covalent anchoring stability of CA on the carbon matrix.

**Figure 6:**
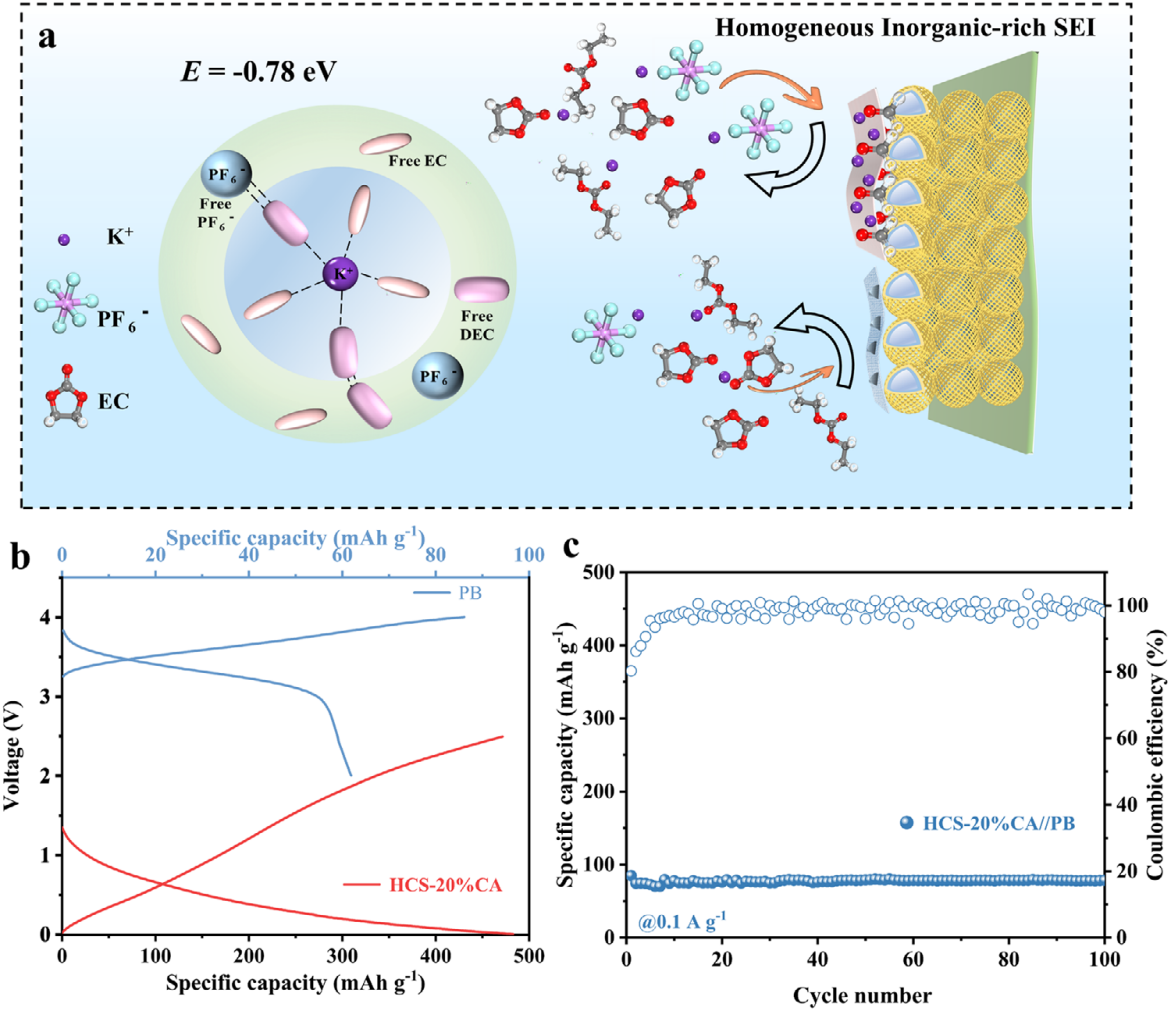
a) Structural model of interfacial solvation and diagram SEI formation in the ester‐based electrolyte. b) Charge/discharge curves of the HCS‐20%CA//K half‐cell and PB//K half‐cell. c) Cycling stability of the HCS‐20%CA //PB full cell at a current density of 0.1 A g^−1^.

To investigate the beneficial effect of C═O groups regarding the formation of the SEI and potassium‐ion storage, theoretical calculations were performed. These structurally anchored functionalities demonstrate superior performance metrics in both interfacial chemistry modulation and K^+^ storage mechanisms when systematically compared with conventional oxygen‐containing groups (e.g., carboxyl (─COOH) and hydroxyl (─OH) functionalities), as evidenced by theoretical calculations. Theoretical calculations (Figure S21, Supporting Information) demonstrate that C═O exhibits the strongest adsorption energy toward KPF₆ compared to ═COOH and ═OH groups. Specifically, while dissociated ═OH and ═COOH groups carry negative charges (O═ and COO─, respectively), leading to electrostatic repulsion toward PF₆⁻, the undissociated C═O maintains polarity without charge accumulation, enabling stronger affinity for KPF₆.^[^ ^45^ ^]^ When hard carbon materials are surface‐modified with oxygen‐containing functional groups, they can alter the arrangement of cationic solvents on the electrode surface. This modification changes the de‐solvation structure of the electrolyte.^[^ ^46^ ^]^ Theoretical calculations indicate that C═O exhibits the strongest adsorption energy toward KPF₆, promoting the preferential reduction of PF₆^−^ in the electrolyte. This process facilitates the formation of a stable inorganic solid electrolyte interface (SEI) layer. In addition, we developed an interface model for K^+^ de‐solvation and elucidated how these functional groups affect the de‐solvation process of K^+^, and thus the electrode performance, on a molecular scale. This phenomenon facilitates the preferential decomposition of the salt into inorganic species, thereby enhancing cycling stability. To further evaluate the practical application potential of HCS‐20%CA, we used Prussian blue type cathode for full‐cell assembly, and the charge/discharge curve of PB half‐cell (**Figure** **6**b), and the capacity is 78.6 mAh g^−1^ after 100 cycles of 0.1 A g^−1^(Figure 6c).

## 3 Conclusion

In summary, the introduction of abundant C═O bonds by grafting caffeic acid on hard carbon microspheres significantly improves the multiplicity performance of PIBs. Combining in situ EIS and XPS etching, it is demonstrated that the presence of C═O promotes the formation of the inorganic‐rich SEI, e.g., KF, which originates from a stronger affinity of C═O moieties on salt, thus achieving excellent interfacial stability and ion diffusion kinetics. Moreover, C═O moieties can reversibly adsorb K‐ions, thus resulting in a high capacitive contribution. As a result, the optimized sample delivers an excellent reversible capacity of up to 462.7 mAh g^−1^ (0.1 A g^−1^ over 50 cycles) and rate performance (321.8 mAh g^−1^ at 2 A g^−1^). Besides that, prolonged cycle life is also realized at 2 A g^−1^ over 2000 cycles in ester‐based electrolytes.

## Conflict of Interest

The authors declare no conflict of interest.

## Supporting information



Supporting Information

## Data Availability

Research data are not shared.
