# Supplementary material for: Carbonyls Mediated Dual‐Function Enables High‐Performance Carbon Anodes in Ester‐based Electrolyte
Source: Adv Sci (Weinh). 2025 Jun 20;12(34):e03954. doi: 10.1002/advs.202503954 (PMC12442670; doi:10.1002/advs.202503954)
Supplement: Supplementary file 1 — Supporting Information [file ADVS-12-e03954-s001.doc]

***Supporting information for***

**Carbonyls Mediated Dual-Function Enables High-Performance Carbon Anodes in Ester-based Electrolyte**

Ziyu Wu, Fei Yuan, Siyu Wu, Di Zhang, Qiujun Wang, Qujiang Sun, Zhaojin Li*, Wei Wang, Bo Wang*

Z. Wu, F. Yuan, S. Wu, D. Zhang, Q. Wang, Q. Sun, Z. Li, B. Wang

Hebei Key Laboratory of Flexible Functional Materials

School of Materials Science and Engineering

Hebei University of Science and Technology

Shijiazhuang 050000, China

E-mail: [wangbo1996@gmail.com](mailto:wangbo1996@gmail.com) (B. W), zjli11s@alum.imr.ac.cn (Z. L)

W. Wang

School of Metallurgical and Ecological Engineering

University of Science and Technology Beijing

Beijing 100083, China

**Experimental Section**

***Synthesis of HCS:*** HCS was synthesized by using SiO_2_ as a template. In brief, the silicon source (tetrapropoxysilane) was added to a mixture solution of ethanol, deionized water and ammonia under stirring at room temperature. After 5 minutes, the resorcinol and formaldehyde were added into the solution and stirred for 24 h, and then centrifugally washed several times. The obtained precursor was dried overnight at 60 ℃, followed by carbonized in Ar atmosphere at 800℃ for 3 h with a heating rate of 5 ℃/min to obtain SiO_2_@C. After removing the SiO_2_ template with a 10 *wt*% HF solution under stirring overnight, the HCS was collected by centrifugation and washed with deionized water and ethanol several times.

***Synthesis of HCS-20%CA:*** First, 100 mg HCS was dispersed in 50 mL deionized water under stirring for 0.5 h. Then, different ratios of caffeic acid (CA) were gradually added into the above solution and continuous stirring for 1 h. The mass ratios of CA versus HC were 20%. The solution was then heated at 80 ℃ under constant stirring until the water was evaporated. The dried HC-CA powders were then transferred to a vacuum oven and treated at 80 ℃ for 12 h to induce dehydration process, and the corresponding product was denoted as HCS-20%CA. As a comparison, the control sample was also prepared in the same way as HCS-20%CA only changing the ratio of CA, and the corresponding product was denoted as HCS-10%/20%30%CA.

***Synthesis of*** ***Prussian blue*:** Dissolve 2 mmol of FeCl_3_·3H_2_O in 40 mL of deionized water to form solution A, and 1 mmol K_4_Fe(CN)_6_·3H_2_O was dissolved in 160 mL deionized water to form solution B, and solution A was added to solution B to produce blue precipitation and form solution C. In order to make the reaction happen evenly, the mixed solution C was placed under magnetic force. The stirring is carried out under the mixer, and the stirring temperature is set to 25℃ and the rotating speed is 800 r·min^-1^. After stirring, let it rest for 24 h, and then centrifuge to obtain the dark blue product Prussian blue. The blue precipitate will be washed with the mixed solution of deionized water and alcohol until the waste liquid is no longer cloudy, and then dried in a vacuum drying oven at 80 ℃ for 24 hours to obtain the final cathode material Prussian Blue (PB).

***Materials Characterizations*:** The morphologies were investigated by scanning electron microscopy (SEM, Hitachi SU8010) and transmission electron microscope (TEM, FEI Tecnai G2 F20 S-TWIN). The chemistry information was examined by X-ray diffraction (XRD, Bruker D2 Phaser X with Cu Kα radiation) and Raman spectroscopy (a Kr-Ar ion laser at 532 nm produced by Spectra-Physics Beamlok 2060-RS laser combining Symphony CCD-1LS detection system). The nitrogen adsorption-desorption isotherms were measured with a Quantachrome Autosorb AS-6B system. Then, Fourier-transform infrared (FT-IR) spectrum was obtained using a Thermo Scientific Nicolet iS10 spectrometer. The near surface chemical state of different elements was also tested via X-ray photoelectron spectroscopy (XPS, ESCALAB 250 Xi).

***Electrochemical Measurements*:** The anodes were prepared by mixing active material, acetylene black (AB), and polyvinylidene ﬂuoride (PVDF) with a mass ratio of 7:2:1. The slurry was cast onto the copper foil and dried at 80 °C under vacuum for 12 h. Coin cells (CR2032) were assembled with potassium foil as the counter/reference electrode, prepared anodes with a mass loading of ca. 1.5 mg cm^−2^ as work electrode, a glass-ﬁber as separator, and by using 0.8 M KPF6 dissolved in ethylene carbonate (EC)/diethyl carbonate (DEC) (1:1 by volume) as an electrolyte in argon-ﬁlled glove box. The galvanostatic tests of cycle and rate performance were tested by multi-channel land battery test system (LAND-CT2001A) in the ﬁxed voltage window from 0.01 V to 2.5 V versus K^+^/K at room temperature. Cyclic voltammetry (CV) was conducted by a Gamry electrochemical workstation with a scan rate of 0.1 mV s^−1^. Electrochemical impedance spectroscopy (EIS) was measured on a Gamry electrochemical workstation over the frequency range from 0.01 to 10^5^ Hz (amplification voltage: 5 mV). The galvanostatic intermittent titration technique (GITT) measurements were tested on a LAND-CT2001A battery testing system by alternating the current density of 100 mA g^-1^ for 30 min with rest intervals for 3 h in the voltage range of 0.01-2.5 V.

**

**

**Fig. S1.** Atomic ratio of HCS and HCS-20%CA obtained by corresponding element mapping.


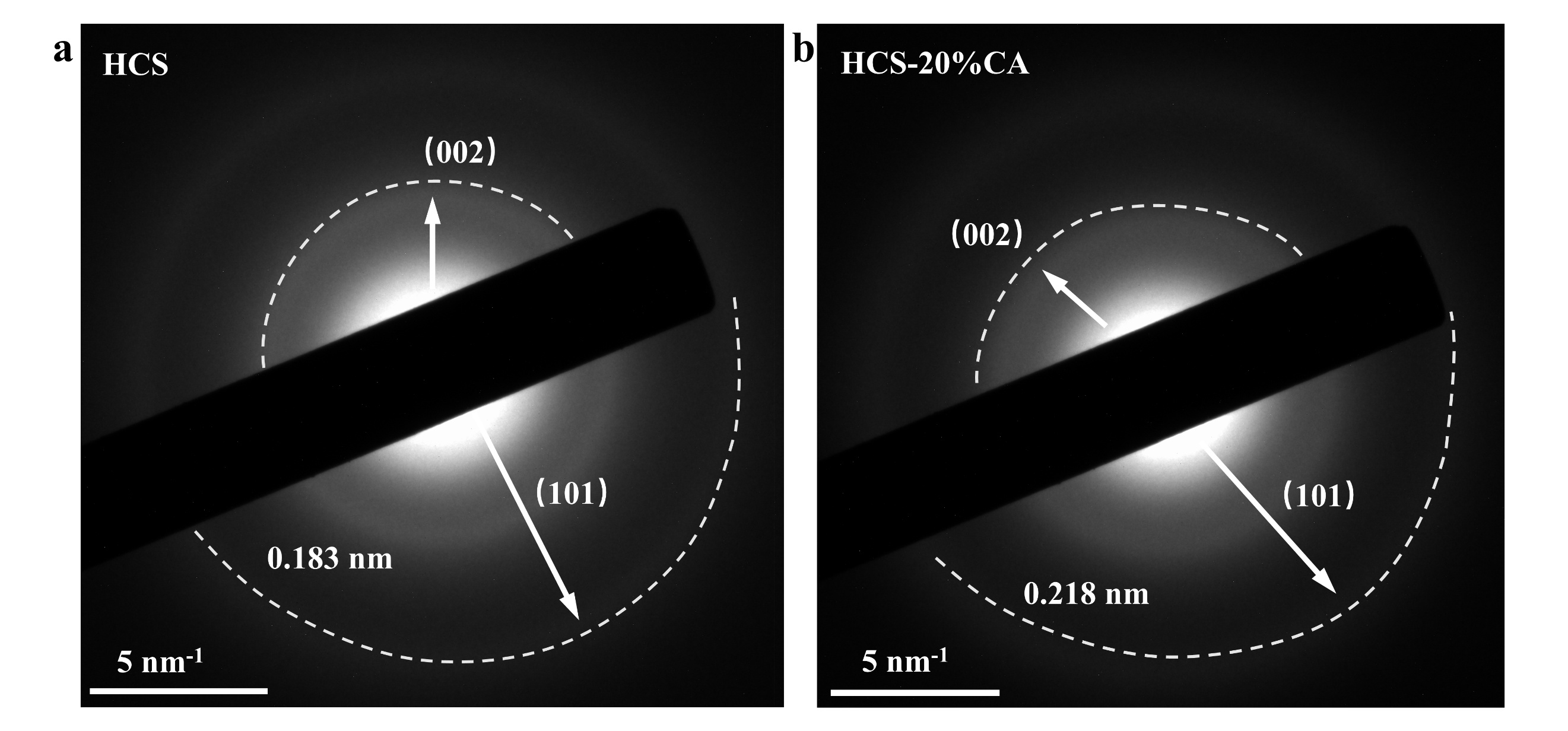


**Fig. S2** SAED images of (a) HCS and (b) HCS-20%CA

**

**

**Fig. S3.** XRD spectra of HCS-10%CA and HCS-30%CA.





**Fig. S4.** Raman spectra of HCS-10%CA, HCS-20%CA and HCS-30%CA.





**Fig. S5.** FT-IR spectra of HCS, HCS-10%CA, HCS-20%CA and HCS-30%CA.





**Fig. S6.** XPS spectra of HCS, HCS-10%CA, HCS-20%CA and HCS-30%CA.

**
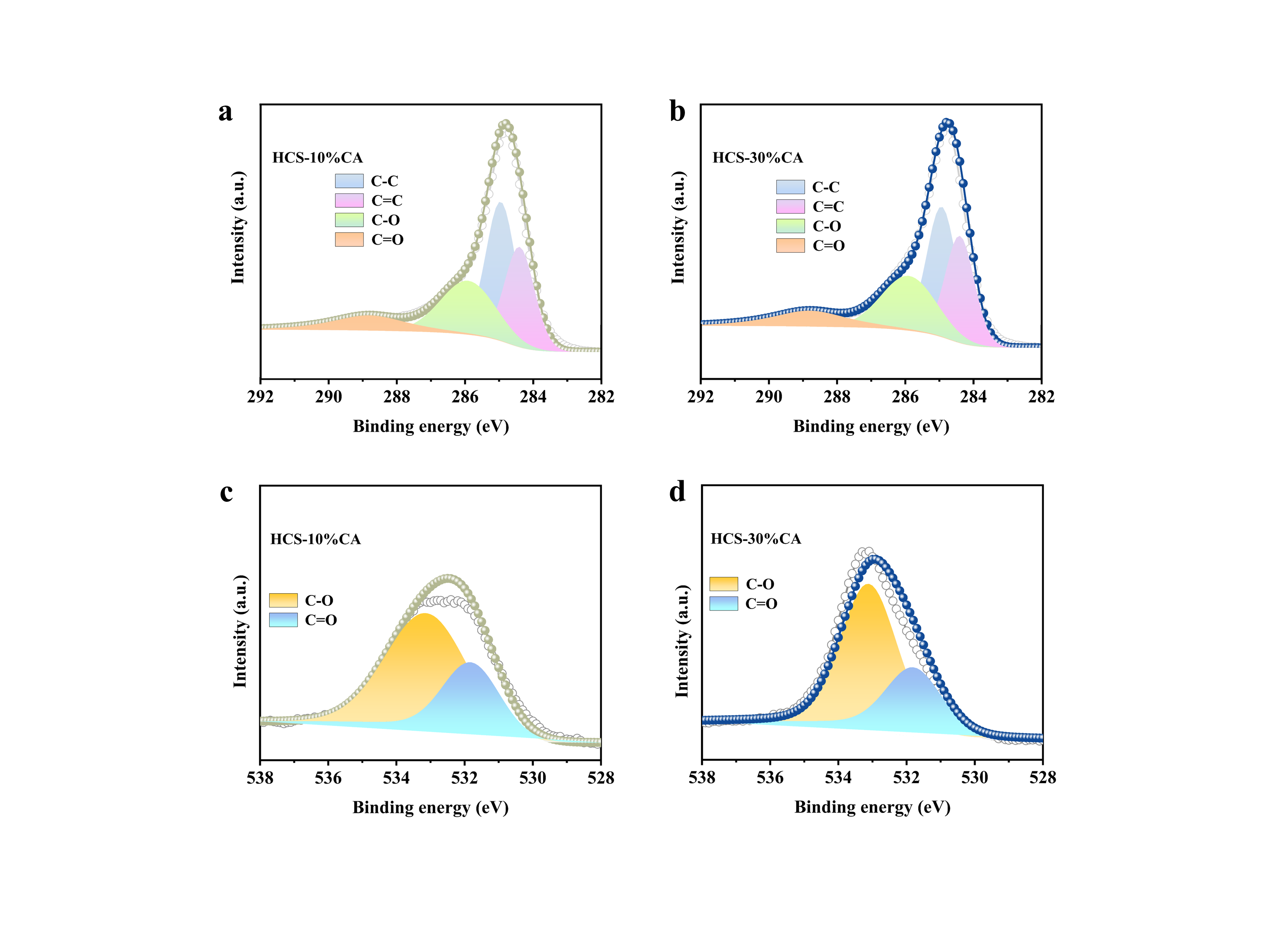
**

**Fig. S7** High-resolution XPS spectra of (a, b) C 1s and (c, d) O 1s for HCS-10%CA and HCS-30%CA.

**

**

**Fig. S8.** C=C, C-C, C-O, and C=O proportion of HCS-10%CA and HCS-30%CA.





**Fig. S9.** C-O, and C=O proportion of HCS, HCS-10%CA, HCS-20%CA and HCS-30%CA.

**
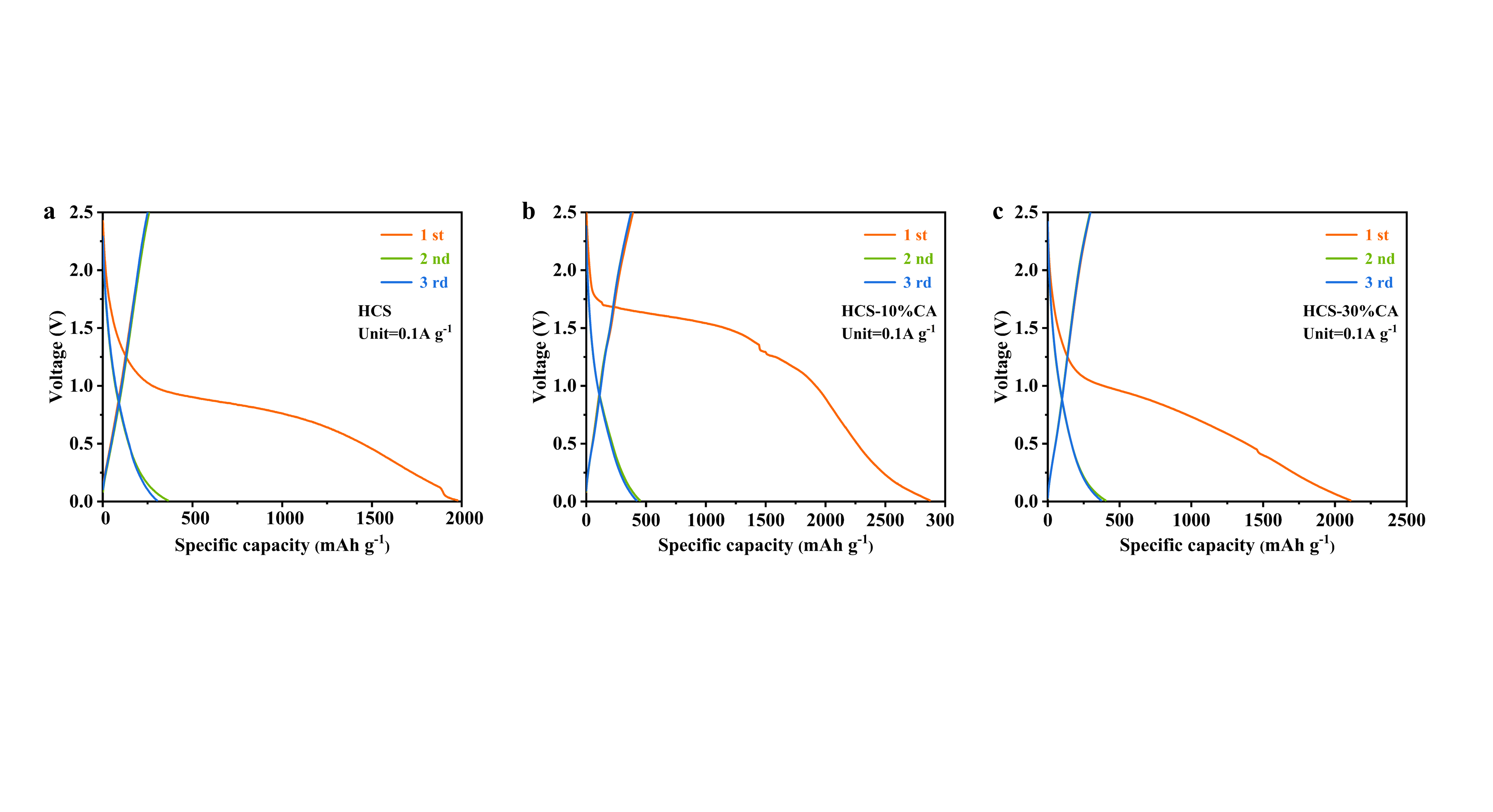
**

**Fig. S10.** Charge and discharge curve of HCS, HCS-10%CA and HCS-30%CA at 0.1A g^-1^.

**

**

**Fig. S11.** Cycle performance of HCS, HCS-10%CA, HCS-20%CA and HCS-30%CA at 0.5 A g^-1^.

**

**

**Fig. S12.** Charge and discharge curve of HCS at current densities.

**
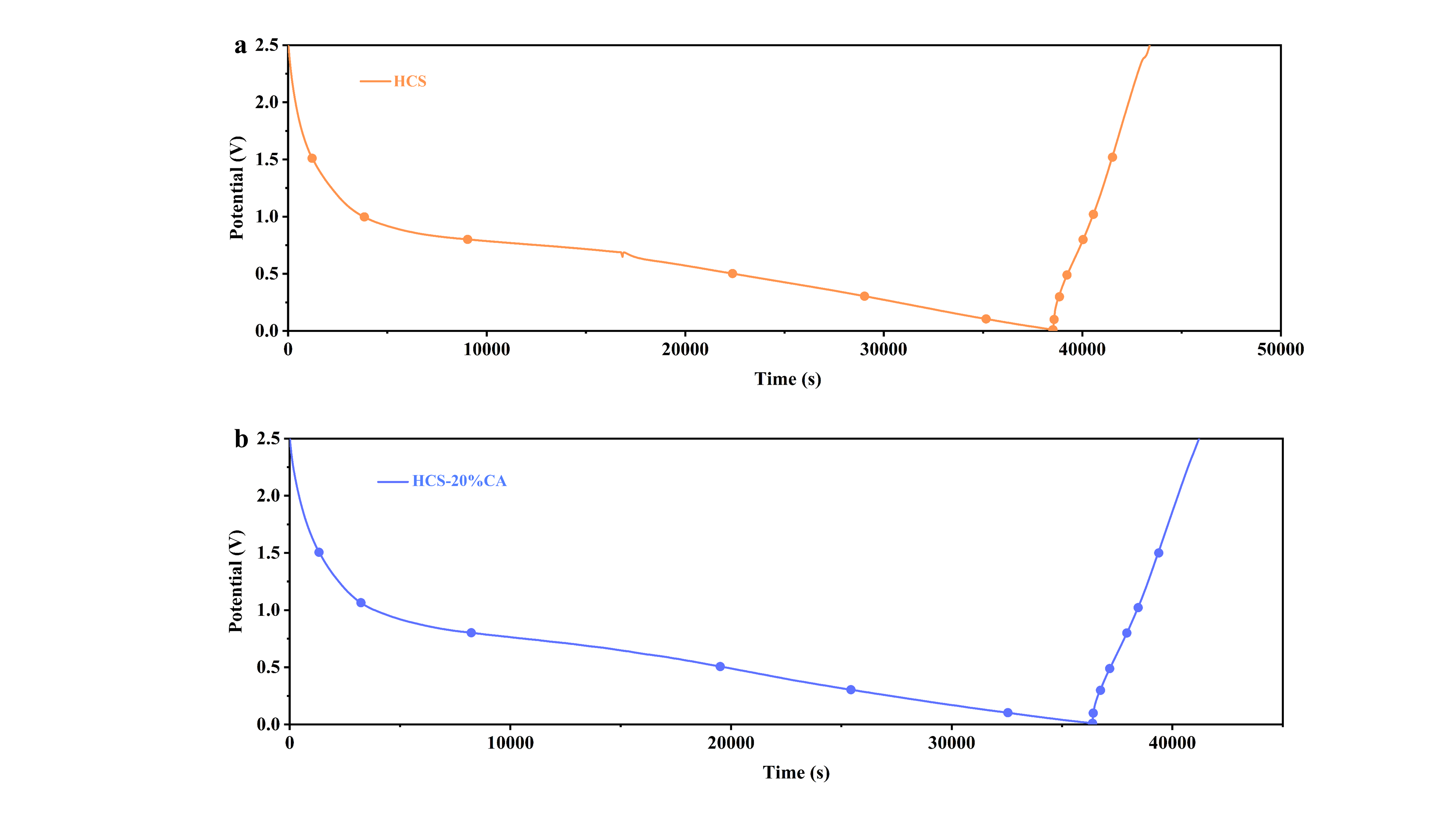
**

**Fig. S13.** GCD profile of (a) HCS and (b) HCS-20%CA during the in-situ EIS measurement.


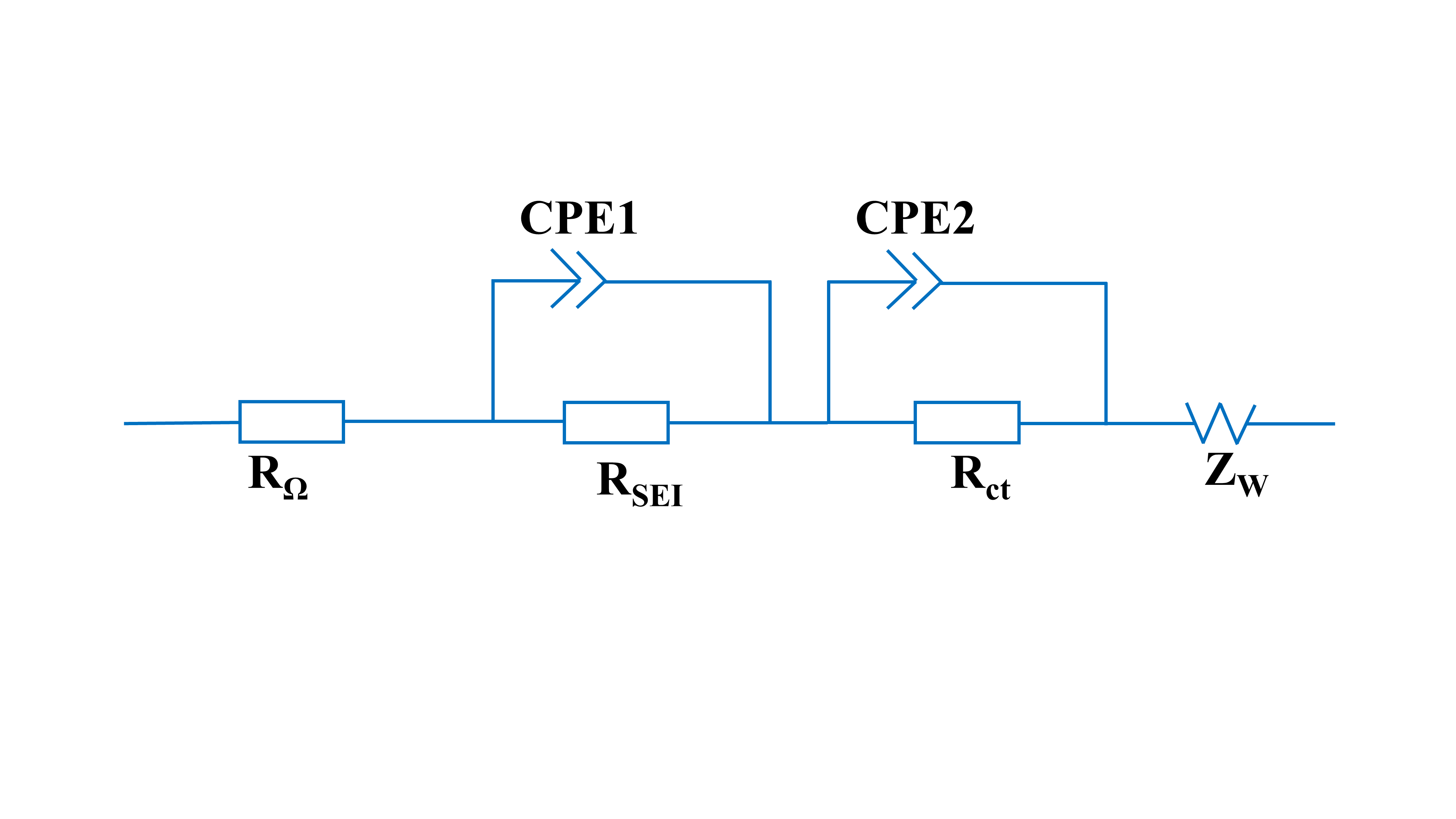


**Figure S14.** Eequivalent circuit for EIS fitting.


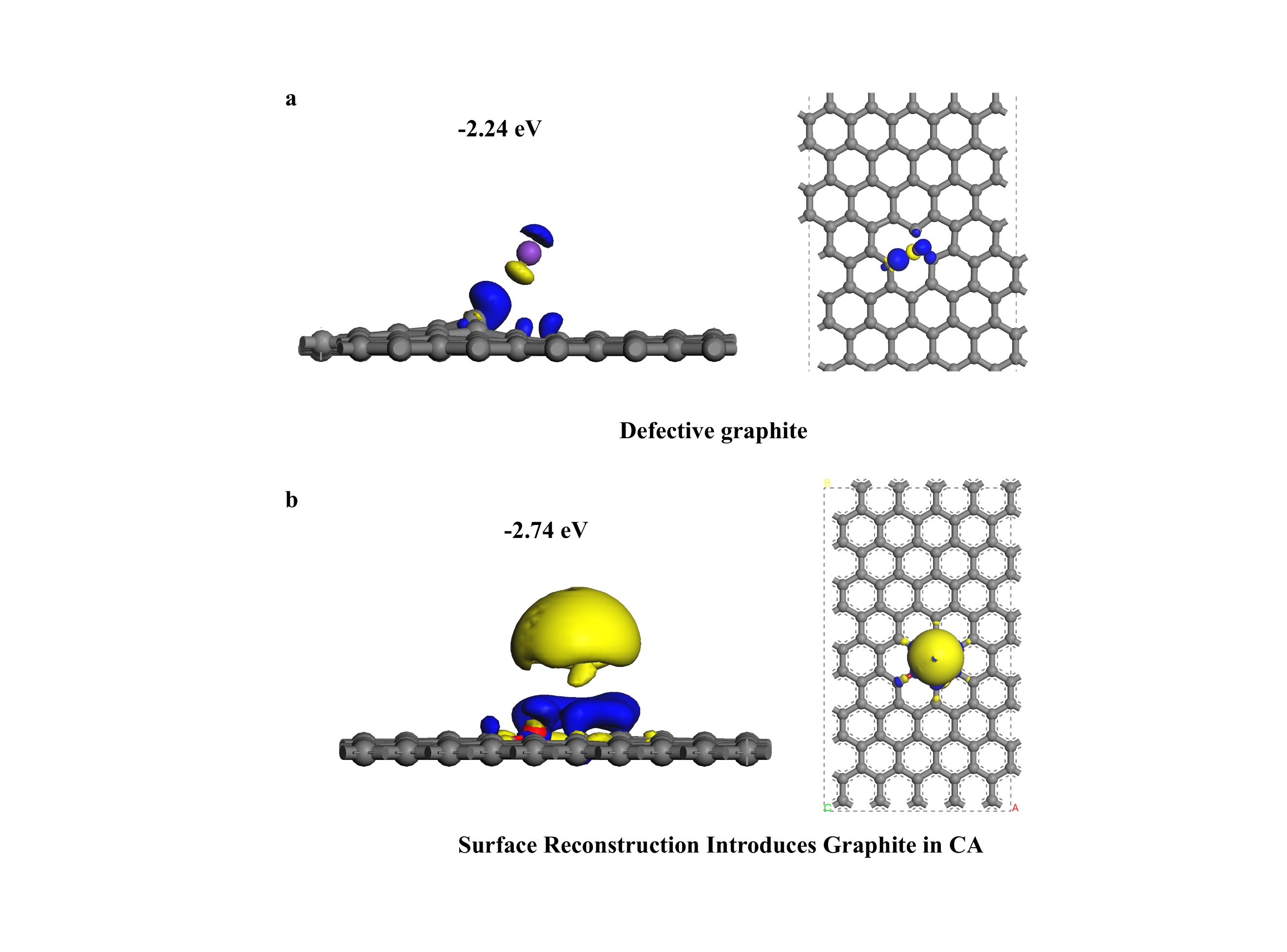


**Fig. S15.** The optimized configurations of (a) defective graphene and (b) surface reconstruction introducing CA adsorption energies for K-ion on their corresponding surfaces.


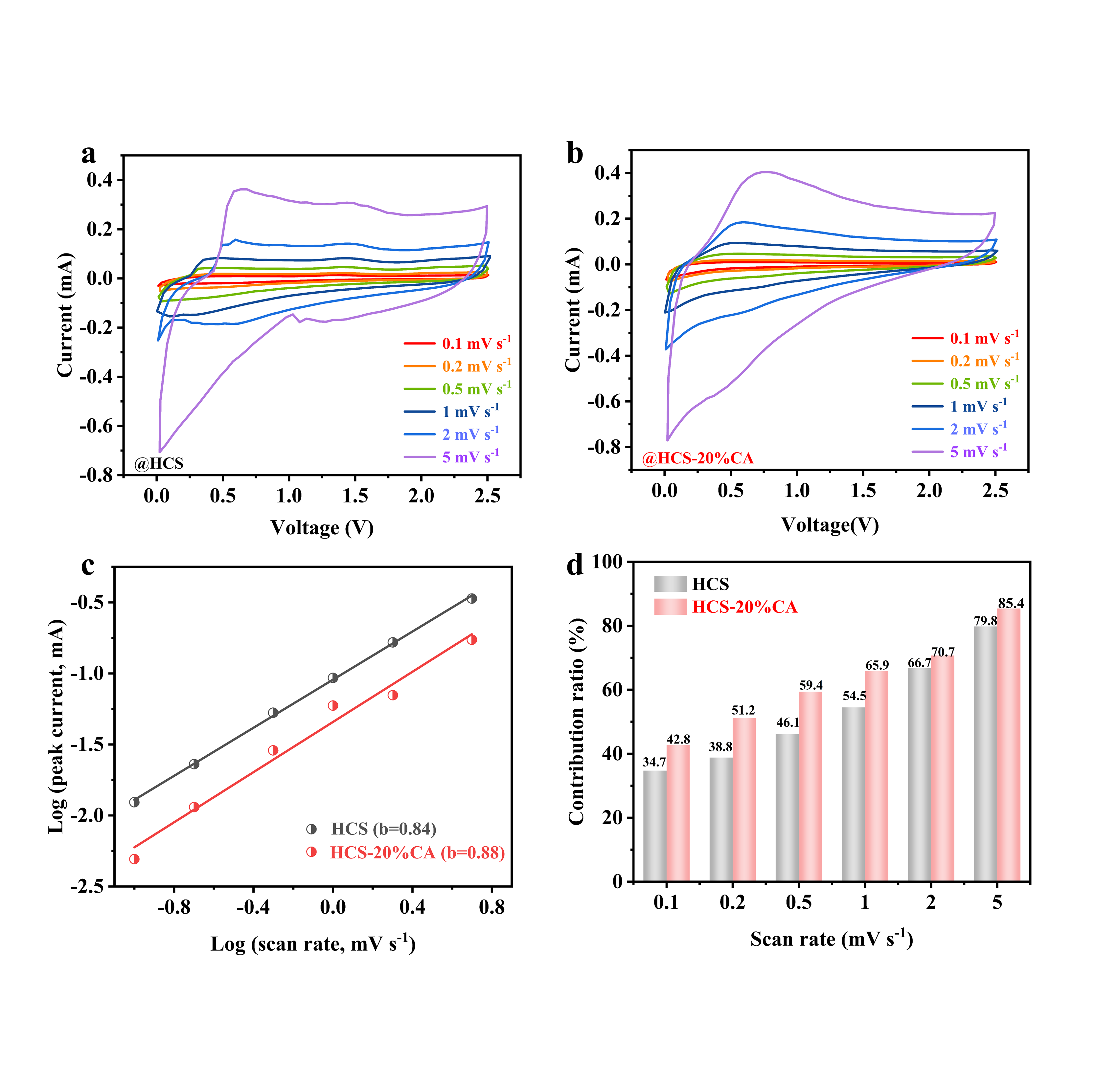


**Fig. S16.** (a) CV profiles of HCS and (b) CV profiles of HCS-20%CA at different scan rates. (c) The b values fitting plots of HCS and HCS-20%CA. (d) the calculated capacitive contribution of H HCS-20%CA.


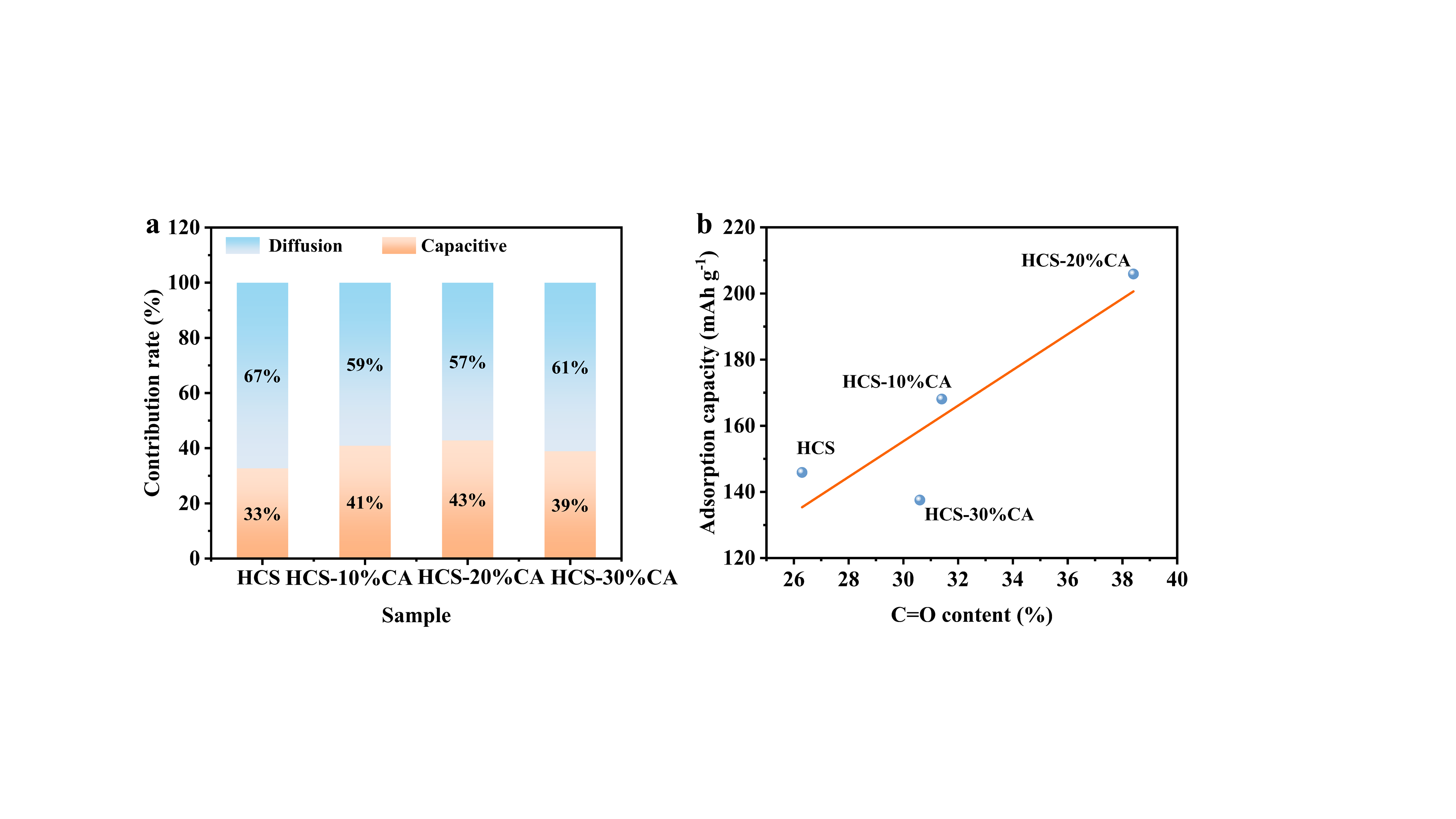


**Fig. S17 (**a) Diffusion contribution at 0.1 mV s^-1^, (b) diffusion capacity at 0.1 A g-1, versus C=O functional group content of HCS, HCS-10%CA, HCS-20%CA and HCS-30%CA at 0.5 A g^-1^.





**Fig. S18.** GITT profiles at 0.1 A/g of HCS and HCS-20%CA.

**

**

**Fig. S19.** Schematic of the calculation of diffusion coefficient using GITT technique.

The ionic diffusion coefficient in HCS and HCS-20%CA electrodes can be determined by solving Fick’s second law with the following equation

D=
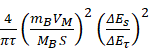


where m_B_ is electrode active mass; M_B_ is the molar mass of the electrode material for carbon; V_M_ is the molar volume of hard carbon; S is the geometric area of the electrode; M_B_/V_M_ is obtained from the density of HCS and HCS-20%CA.


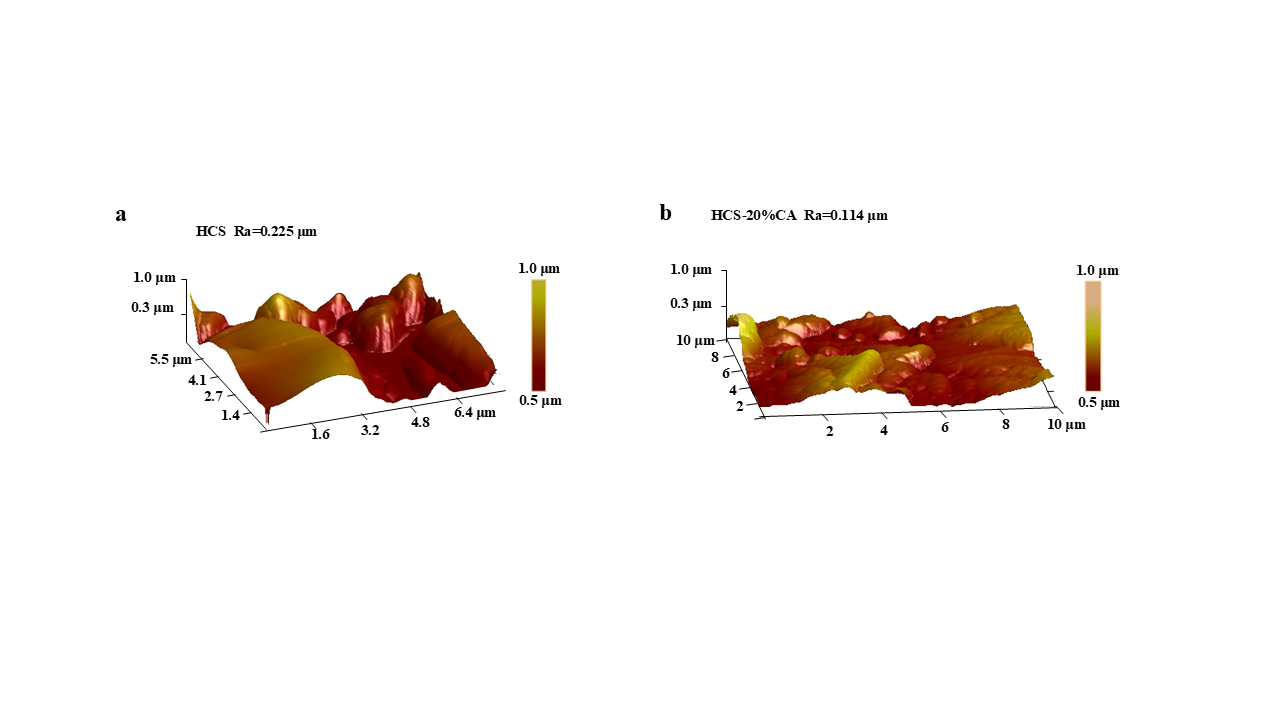


**Fig S20**. 3D topographical AFM images of (a) HCS and (j)HC-20%CA


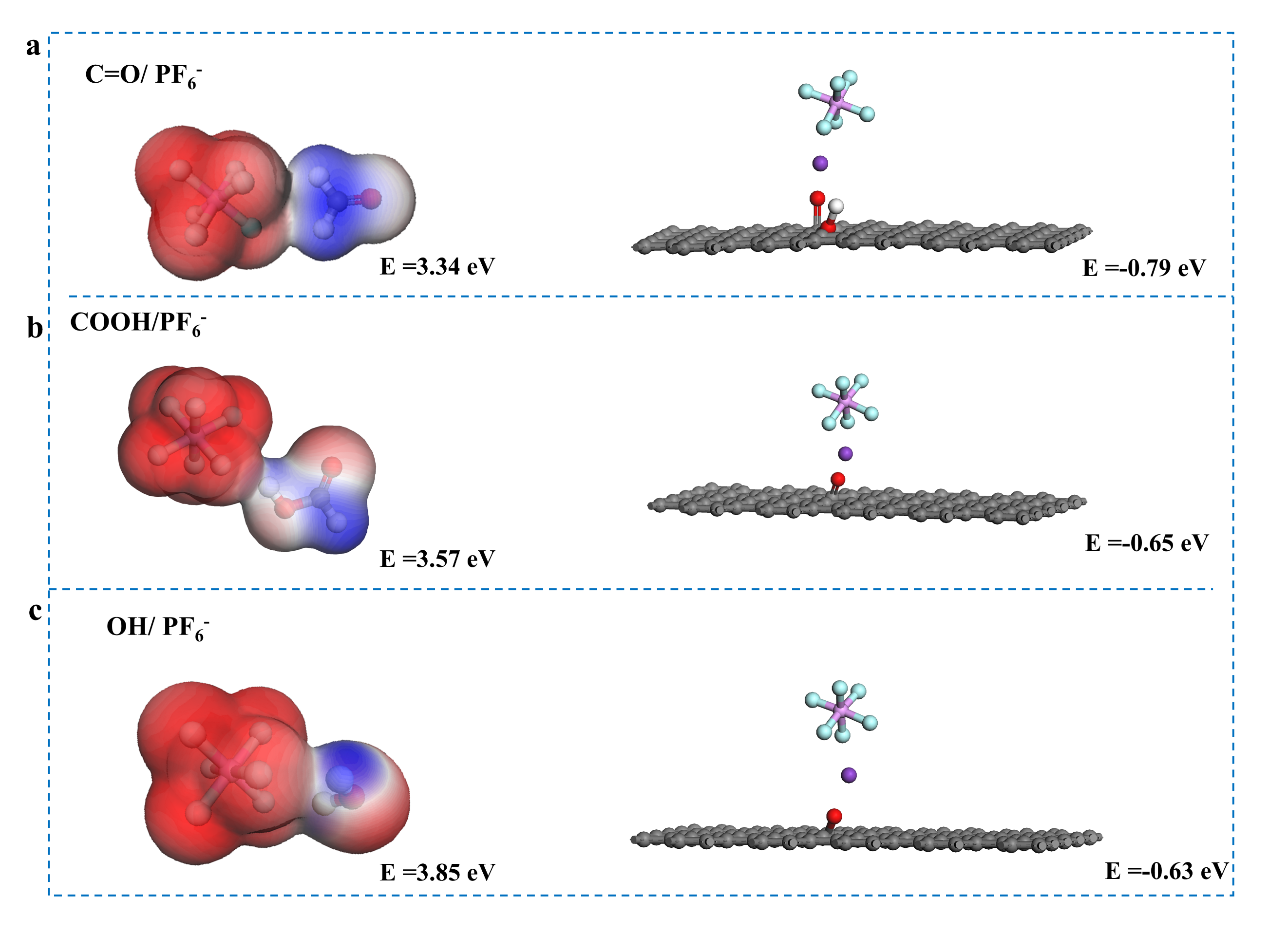


**Fig. S21** Binding energies of C=O/COOH and OH for KPF_6_.

**Table S1.** The cycling performance comparison of HCS-20%CA and previously reported state-of-the-art carbonaceous materials for PIBs.

| **Materials** | **Electrolyte** | **Voltage**  **range(V)** | **Cycling performance** | | | **Ref.** |
| --- | --- | --- | --- | --- | --- | --- |
|  |  |  | **Current**  **density (mA g^−1^)** | **Cycle**  **number** | **Capacity**  **(mAh g^−1^)** |  |
| **HCS-20%CA** | **0.8M KPF_6_ DEC:EC**  **(1:1, v/v)** | **0.01-2.5** | **2000** | **2000** | **216** | **This work** |
| NOSHC-101-500-1 | 1 M KFSI /EC-DEC (1:1, v/v) | 0.01-3.0 | 1000 | 1000 | 210 | Ref. 1 |
| PSC\PHC-0.5 | 0.8 M KPF_6_/EC-PC  (1:1, v/v) | 0.01-3.0 | 1000 | 2000 | 166 | Ref. 2 |
| VCA-5 | 0.8M KPF_6_ DEC:EC  (1:1, v/v) | 0.01-2.5 | 150 | 1000 | 151 | Ref. 3 |
| HAT-COOK (CMC) | 1 M KFSI/EC-DMC  (1:1, v/v) | 0.01-3.0 | 500 | 500 | 143 | Ref. 4 |
| S-PC | 1 M KFSI / EC-DEC  (1:1, v/v) | 0.01-3.0 | 1000 | 3000 | 204 | Ref. 5 |
| SPCS | 1 M KFSI / DME  (100%, v) | 0.01-3.0 | 2000 | 1500 | 129.7 | Ref. 6 |
| CCHC | 1M KPF_6_ / DME  (100%, v) | 0.01-3.0 | 2000 | 1000 | 116.9 | Ref. 7 |
| HC-1300 | 0.8 M KPF_6_/EC-DMC  (1:1, v/v) | 0.01-2.5 | 1000 | 1000 | 141 | 0.01-2.5 |
| SnSb@NC | 0.5 M KPF_6_/DME  (100%, v) | 0.01-2.2 | 500 | 200 | 185.8 | Ref. 9 |
| CND | 1 M KFSI/THF | 0.01-3.0 | 50 | 50 | 200 | Ref. 10 |

**Table S2.** XPS binding energy and peak assignment for the SEI components formed on the

HCS-20%CA anode.

|  | 0 s (%) | 50s (%) | 100s (%) | Assignment | Species |
| --- | --- | --- | --- | --- | --- |
| C | 93 | 87.5 | 81.9 | Organic |  |
|  | 7.0 | 12.5 | 18.1 | CO_3_^2-^ |  |
| O | 23.3 | 18.9 | 20.0 | O=C-O |  |
|  | 31.4 | 30.7 | 32.6 | C-O/OH |  |
|  | 25.7 | 27.2 | 24.0 | C=O/K-O |  |
|  | 19.6 | 23.1 | 23.4 | K2CO3 |  |
| F | 62.9 | 55.9 | 52.5 | C-F | -CF_3_ |
|  | 37.1 | 44.1 | 47.5 | K-F | KF |

**Table S3.** XPS binding energy and peak assignment for the SEI components formed on the HCS anode.

|  | 0s (%) | 50s (%) | 100s (%) | Assignment | Species |
| --- | --- | --- | --- | --- | --- |
| C | 98.3 | 89.1 | 88.9 | Organic |  |
|  | 1.7 | 10.8 | 11.1 | CO_3_^2-^ |  |
| O | 16.1 | 19.8 | 21.5 | O=C-O |  |
|  | 44.0 | 29.4 | 30.7 | C-O/OH |  |
|  | 20.1 | 27.5 | 25.3 | C=O/K-O |  |
|  | 19.8 | 23.3 | 22.5 | K2CO3 |  |
| F | 72.0 | 66.7 | 64.4 | C-F | -CF_3_ |
|  | 28.0 | 33.3 | 35.6 | K-F | KF |

**Table S4.** Comparison of C=O content in O 1s of initial and post-cycling samples of HCS and HCS-20% CA

| **Materials** | C=O (Initial sample) | C=O (After the cycling) |
| --- | --- | --- |
| HCS | 26.6% | 20.1% |
| HCS-20%CA | 38.4% | 25.7% |

**References**

[1] Lu X, Zhou J, Huang L, Peng H, Xu J, Liu G, Shi C, Sun Z. *Adv. Energy Mater.* **2024**, *12,* 2303081.

[2] Y. Li, A. Zhu, G. Peng, J. He, H. Li, D. Jia, J. Qiu, X. He, *J. Energy Chem*. **2025**, *103,* 97–105.

[3] Wang J, Xu Z, Eloi JC, Titirici MM, Eichhorn SJ. *Adv. Funct. Mater*. **2022**, *32*, 2110862.

[4] J. Zou, C. Fu, Y. Zhang, K. Fan, Y. Chen, C. Zhang, G. Zhang, H. Dai, Y. Cao, J. Ma, C. Wang, *Adv. Funct. Mater*. **2023**, *33*, 2303678.

[5] Z. Bo, P. Chen, F. Tian, Y. Huang, Z. ZHENG, J. Yan, K.Cen, H. Yang, K. Ostrikov, *Carbon* **2023,** *213*, 118261.

[6] H. Zhang, C. Luo, H. He, H. Wu, L. Zhang, Q. Zhang, H. Wang, M. Wang, *Nanoscale Horiz,* **2020**, *5*, 8950903.

[7] H. Dai, Z. Zeng, X. Yang, M. Jiang, Y. Wang, Q. Huang, L. Liu, L. Fu, P. Zhang, Y. Wu, *Carbon,* **2021**, *179*,60-67.

[8] H. Ou, B. Pei, Y. Zhou, M. Yang, J. Pan, S. Liang, X. Cao, *Small Methods.* **2024,** *9,* 2400839.

[9] Z. Wang, K. Dong, D. Wang, S. Luo, Y. Liu, Q. Wang, Y. Zhang, A. Hao, C. Shi, N. Zhao, *J. Mater. Chem. A*, **2025**, *13*, 5777-5788.

[10] Z. Yu, Q. Liu, C. Chen, Y. Zhu, B. Zhang, *J. Power Sources*, **2023**, *557*, 22259.
